# Renovascular hypertension in children

**DOI:** 10.1186/s42155-020-00176-5

**Published:** 2021-01-07

**Authors:** Premal Amrishkumar Patel, Anne Marie Cahill

**Affiliations:** 1 Interventional Radiology, Radiology Department, Great Ormond Street Hospital for Children, Great Ormond Street, London, WC1N 3JH UK; 2 Interventional Radiology, Department of Radiology, Children’s Hospital of Philadelphia, Philadelphia, PA USA

**Keywords:** Paediatric interventional radiology, Renal artery stenosis, Fibro-muscular dysplasia, Mid-aortic syndrome

## Abstract

Paediatric hypertension, defined as systolic blood pressure > 95th percentile for age, sex and height is often incidentally diagnosed. Renovascular hypertension (RVH) is responsible for 5–25% of hypertension in children. Renal artery stenosis and middle aortic syndrome can both can be associated with various conditions such as fibromuscular dysplasia, Williams syndrome & Neurofibromatosis type 1. This paper discusses the approaches to diagnosis and interventional management and outcomes of renovascular hypertension in children. Angiography is considered the gold standard in establishing the diagnosis of renovascular disease in children. Angioplasty is beneficial in the majority of patients and generally repeated angioplasty is considered more appropriate than stenting. Surgical options should first be considered before placing a stent unless there is an emergent requirement. Given the established safety and success of endovascular intervention, at most institutions it remains the preferred treatment option.

## Background

Paediatric hypertension is defined as systolic blood pressure > 95th percentile for age, sex and height (National High Blood Pressure Education Program Working Group on High Blood Pressure in Children and Adolescents 2004). Despite guidelines from USA and Europe strongly recommending regular blood pressure screening in children (Tullus 2018), blood pressure in children is not often measured therefore, hypertension is usually an incidental diagnosis in childhood. Early identification and management of hypertension can help reduce the risk of long term cardiac and cerebrovascular events and renal impairment (Bartosh and Aronson 1999; Zhu et al. 2014).

RVH is hypertension resulting in alteration of the renin-angiotensin mechanism secondary to a lesion reducing blood flow to part or all of one or both kidneys (Lobeck et al. 2018). RVH is responsible for 5–25% of hypertension in children and is potentially amenable to treatment for the purposes of control and in many cases cure (Zhu et al. 2014; Kurian et al. 2013; Meyers et al. 2014; Tullus et al. 2008; Srinivasan et al. 2011). This paper discusses the approaches to diagnosis and interventional management and outcomes of renovascular hypertension in children.

## Associated diseases

RVH can be caused by numerous pathologies both syndromic and non-syndromic. Renal artery stenosis (RAS) and middle aortic syndrome (MAS) can both be associated with various conditions such as fibromuscular dysplasia (FMD), Williams syndrome, Neurofibromatosis type 1, tuberous sclerosis and vasculitidies (Takayasu aorto-arteritis arteritis, Kawasaki disease, polyarteritis nodosa). RVH can also occur as a consequence of radiation vasculopathy, thrombosis and transplant renal artery stenosis. RAS and MAS can occur in many cases as an isolated entity (Lobeck et al. 2018; Roebuck 2008; Sethna et al. 2008).

The First International Consensus on the diagnosis and management of fibromuscular dysplasia defined FMD as “a non-atherosclerotic arterial disease that is characterized by abnormal cellular proliferation and distorted architecture of the arterial wall” (Gornik et al. 2019). The consensus document states that FMD primarily manifests as beaded (multifocal) or focal lesions in medium or small-sized arteries, although can include arterial dissection, aneurysm, and tortuosity (Gornik et al. 2019). The prevalence and manifestation of FMD in children is presently unknown, and although previously thought to be a rare disease, research emerging from patient registries in North America and Europe challenges this notion (Louis et al. 2018). Non-syndromic FMD is thought to be the most common cause of renovascular hypertension in children (Meyers et al. 2014; Tullus et al. 2010). Unifocal FMD is more common than the multifocal variant (Louis et al. 2018). The rate of observance of the classical angiographic appearance of multifocal FMD, the “string of beads” sign in children is unknown (Lobeck et al. 2018) and in our experience it is not often seen. However, in children, histological confirmation is very rarely obtained therefore the diagnosis is usually made by excluding other known causes of renovascular disease such as Takayasu’s arteritis (TA) and neurofibromatosis type 1 (Tullus et al. 2010).

TA is vasculitis involving large arteries which mainly affects young women, but can affect children (Tullus and Roebuck 2018; Forsey et al. 2011; Brunner et al. 2010). It can be a challenging diagnosis to make. There is only one mandatory criteria to make a diagnosis of TA in children, proposed jointly by the European League Against Rheumatism and The Pediatric Rheumatological Society and Pediatric Rheumatology International Trials Organization which is ‘angiographic abnormality not caused by FMD or similar disease’ (Arend et al. 1990; Ozen et al. 2010). TA usually presents generalized symptoms including malaise and weight loss, however around 50% present in a silent “burn out” phase making it appear identical to FMD and further adding to the diagnostic challenge (Tullus and Roebuck 2018).

Besides FMD, neurofibromatosis is the most common cause for renal artery stenosis in children in western countries (Srinivasan et al. 2011). NF1 is a neurogenetic disorder transmitted as an autosomal dominant trait with classic manifestations including café-au-lait macules, skinfold freckling, neurofibromas, brain tumors, iris hamartomas, and characteristic bony lesions. NF1-associated vasculopathies and renovascular hypertension are a little publicized but important manifestation and include abdominal aortic stenosis and osteal and non-osteal renal artery stenosis (Lie 1998).

Williams syndrome is a multisystem disorder with cardiovascular abnormalities, due to elastin deficiency, being the main cause of morbidity and mortality (Collins 2018).

## Renal artery stenosis and middle aortic syndrome

RVH can be caused by an isolated renal artery stenosis (RAS) and/or middle aortic syndrome (MAS). RAS can be uni- or bilateral and has been reported to be bilateral in 24–78% in paediatric series (Lobeck et al. 2018; Tullus et al. 2008). When RAS are bilateral there are often concomitant aortic and other visceral vessel abnormalities (Lobeck et al. 2018). Focal arterial stenoses are most common in children (Vo et al. 2006). Diffuse stenoses are more likely when there is extra-renal disease, syndromes or vasculitidies (Lobeck et al. 2018; Vo et al. 2006). RAS lesions have been reported throughout the renovascular tree, with approximately 25% in the main renal arteries, 50% in 2nd order branches, 12.5% in more distal ‘parenchymal’ branches, and 12.5% in accessory renal arteries (Srinivasan et al. 2011; Vo et al. 2006).

Middle aortic syndrome (MAS) is a clinical condition generated by segmental narrowing of the abdominal or distal descending thoracic aorta (Lobeck et al. 2018; Delis and Gloviczki 2005). Most MAS are idiopathic (64%) with pathogenesis being largely speculative (Rumman et al. 2015). Events occurring around day 25 of fetal development when the two embryonic dorsal aortas fuse and lose their intervening wall to form a single vessel have been hypothesized to be the cause of many cases of MAS (Stanley et al. 2008). Concomitant RAS is seen in around 60% of children with mid aortic syndrome (Rumman et al. 2015). MAS is also associated with intestinal, iliac, carotid, cerebral and brachial arterial stenoses (Zhu et al. 2014; Tullus et al. 2008; Sethna et al. 2008; Rumman et al. 2015; Shroff et al. 2006; Kari et al. 2015; Stanley et al. 2006; Tyagi et al. 1997; Tummolo et al. 2009; Trautmann et al. 2017). Srinivasan et al. (2011) demonstrated in a study of 68 angiograms in children with renovascular hypertension that MAS is most commonly associated with NF1.

## Diagnosis of Renovascular hypertension in children

### Ultrasound

A renovascular cause for hypertension is suggested by ultrasound when a stenosis is directly visualized, a parvus et tardus waveform pattern is seen or there are pathologic age-dependent flow parameters (peak systolic flow greater than 180 or 200 cm/s, acceleration time > 80 ms, renal artery to aortic flow velocity ratio > 3, and difference in resistive index more than 0.05) (Trautmann et al. 2017; Conkbayir et al. 2003; Brun et al. 1997; Krumme et al. 1996). Some authors also consider a significant difference in kidney length (≥1 cm) as an indirect sign of RAS (Trautmann et al. 2017).

Ease and ability to perform ultrasound repeatedly makes this modality the most useful and widely performed baseline investigation. Additionally, it can exclude non-vascular causes for hypertension (such as neuroblastoma or pheochromocytoma) and show discrepant renal lengths and other renal pathologies. Sensitivity and specificity for the detection of renal artery stenosis is thought to be lower than in adult patients (Trautmann et al. 2017), with reported sensitivity for Doppler US of 65–88% and specificity of 83–99% (Trautmann et al. 2017; Brun et al. 1997; Kchouk et al. 1997; Chhadia et al. 2013; Dillman et al. 2017; Castelli et al. 2014). Ultrasound performs best in older children and for detection of aortic stenosis (Roebuck 2008; Dillman et al. 2017). For segmental and subsegmental stenoses, involvement of multiple renal arteries and early branching of the main renal artery ultrasound performs poorly (Dillman et al. 2017).

### Computed tomographic and magnetic resonance angiography

**Fig. 1:**
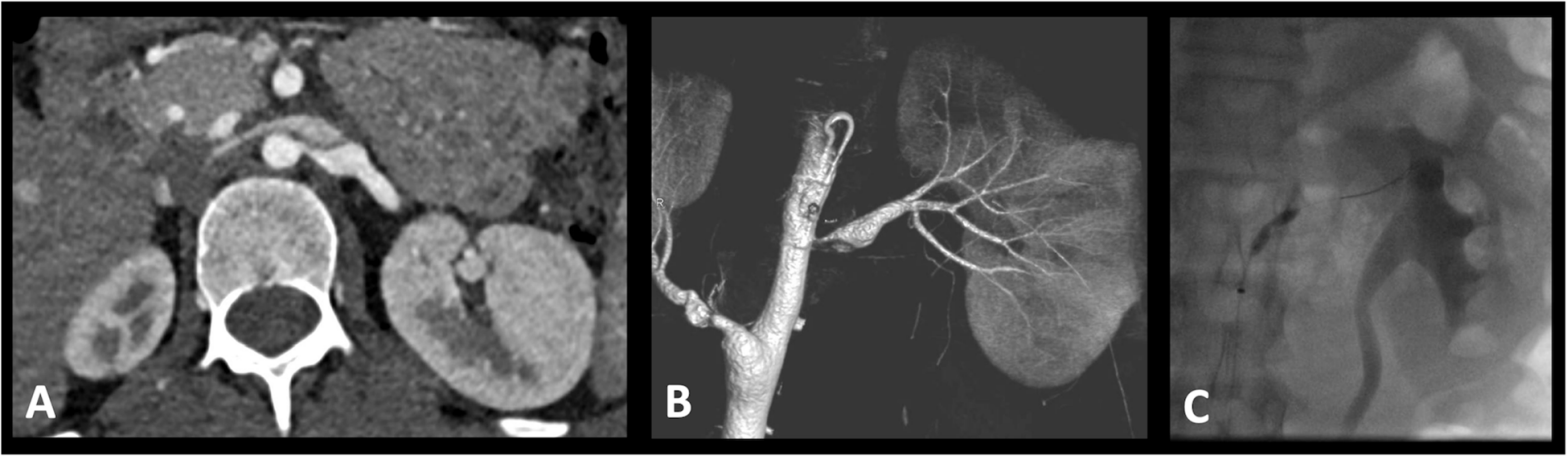
Renal Artery Stenosis Angioplasty. **a** Axial contrast enhanced CT angiogram image demonstrating a left renal artery stenosis with post stenotic dilation. **b** This was confirmed on subsequent rotational angiography and (**c**) at angioplasty by demonstration of a waist on the angioplasty balloon

Computed tomographic angiography (CTA) and magnetic resonance angiography (MRA) are suggestive of a renovascular cause for hypertension when one or more stenoses are visualized as a reduction of the intraluminal diameter (Fig. 1) or if there is the presence of collateral vessels (Trautmann et al. 2017). Similar to ultrasound, in children, CTA and MRA are thought to be adequate for diseases of the aorta but for smaller vessels, especially intraparenchymal branches and in smaller children they are limited (Kurian et al. 2013; Tullus et al. 2008; Louis et al. 2018; Agrawal et al. 2018). In a study evaluating the accuracy of Doppler ultrasound, MRA, and CTA compared to angiography in 127 patients, MRA and CTA were performed in 39 and 34 children, respectively. CTA performed slightly better than MRA with sensitivities of 88% versus 80% and specificity of 81% versus 63% (Trautmann et al. 2017). Trautmann et al. found in their cohort, that CTA missed five cases of renovascular disease, all stenoses were in the main renal artery with additional stenosis in one of the branch renal arteries in one case (Trautmann et al. 2017). MRA missed 10 cases of renovascular disease including stenosis of the main renal artery in six cases, main branch artery in two cases and a segmental artery in two cases (Trautmann et al. 2017). The largest drawback with Truatmann et al.’s (2017) study is that it based on retrospective data using results obtained in many different pediatric radiology departments with variable and possibly non-optimized techniques.

### Angiography

Children who are thought to have a high probability of renovascular disease, for example those with normal body mass index, no family history, elevated renin, hypertension requiring multiple medications should undergo angiography with a view to endovascular treatment regardless of findings of non-invasive imaging (Fig. 1) (Tullus et al. 2008; Louis et al. 2018; Trautmann et al. 2017; Tullus 2011). This is because, in children, angiography is considered the gold standard in establishing the diagnosis of renovascular disease with no non-invasive technique considered capable of excluding renovascular disease (Tullus et al. 2008; Roebuck 2008; Louis et al. 2018; Tullus et al. 2010; Vo et al. 2006; Srinivasan et al. 2010). Louis et al. (2018) found that 28% of vascular lesions in children with hypertension were only detected by angiography. Using the criteria for angiography of poor blood pressure control on two or more medications an abnormality will be seen approximately 40% of the time (Tullus et al. 2008; Vo et al. 2006; Shahdadpuri et al. 2000).

### Intravascular imaging

**Fig. 2:**
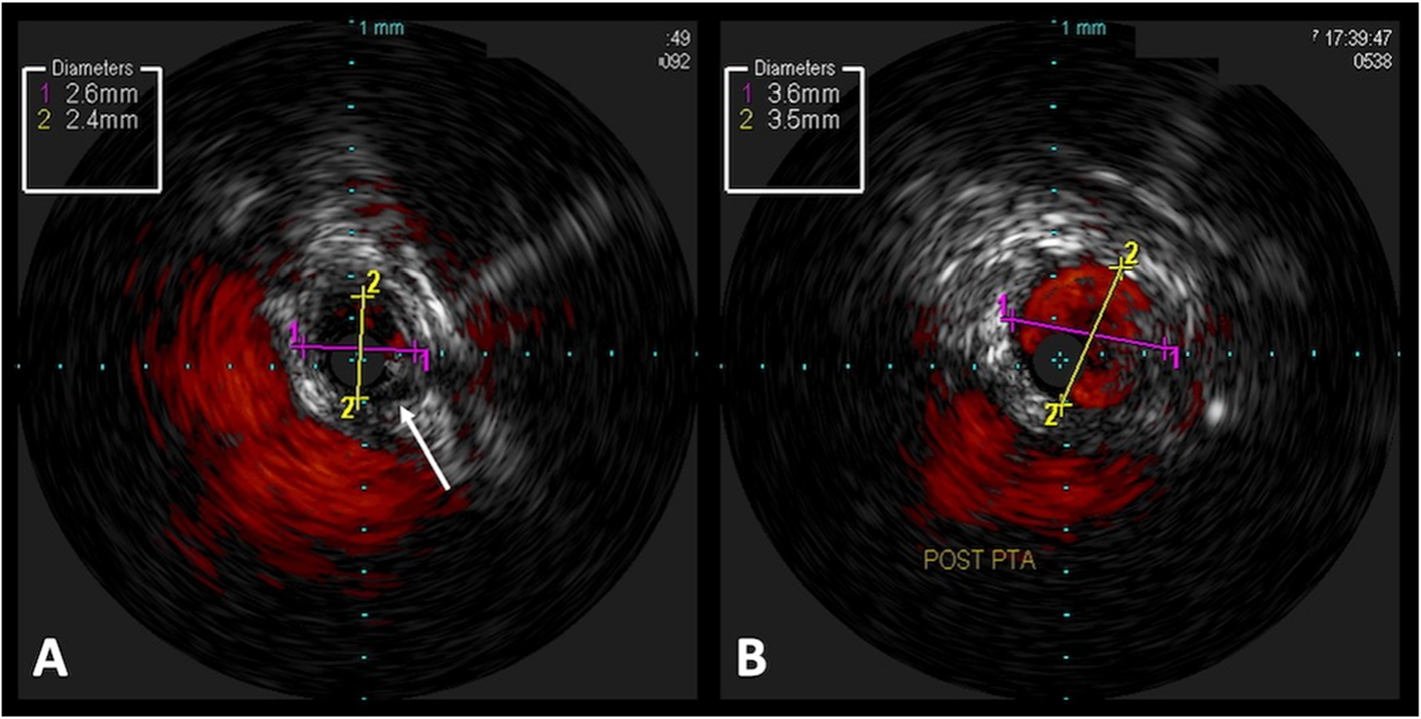
Intravascular ultrasound. **a** IVUS image of severe right renal artery origin stenosis with mural thickening consistent with FMD. **b** Post angioplasty IVUS image of improved flow and vessel diameter right renal artery origin

Angiography allows only visualization of the vessel lumen. High resolution intravascular imaging techniques such as intra-vascular ultrasound (IVUS) (Fig. 2) and optical coherence tomography (OCT) have not been validated in renovascular intervention in children however they may help identify the early arterial wall changes, vessel wall thickness, degree of stenosis, evaluate aneurysmal walls and the results of angioplasty and/or stenting. These techniques have been shown be helpful for directing appropriate stent sizing and identifying acute complications such as dissection and stent malapposition with resultant improved clinical outcomes in adult renal artery and coronary intervention (Maehara et al. 2017; Mizutani et al. 2015; Vijayvergiya et al. 2019; Onoue et al. 2011).

### Contrast US emerging role

**Fig. 3:**
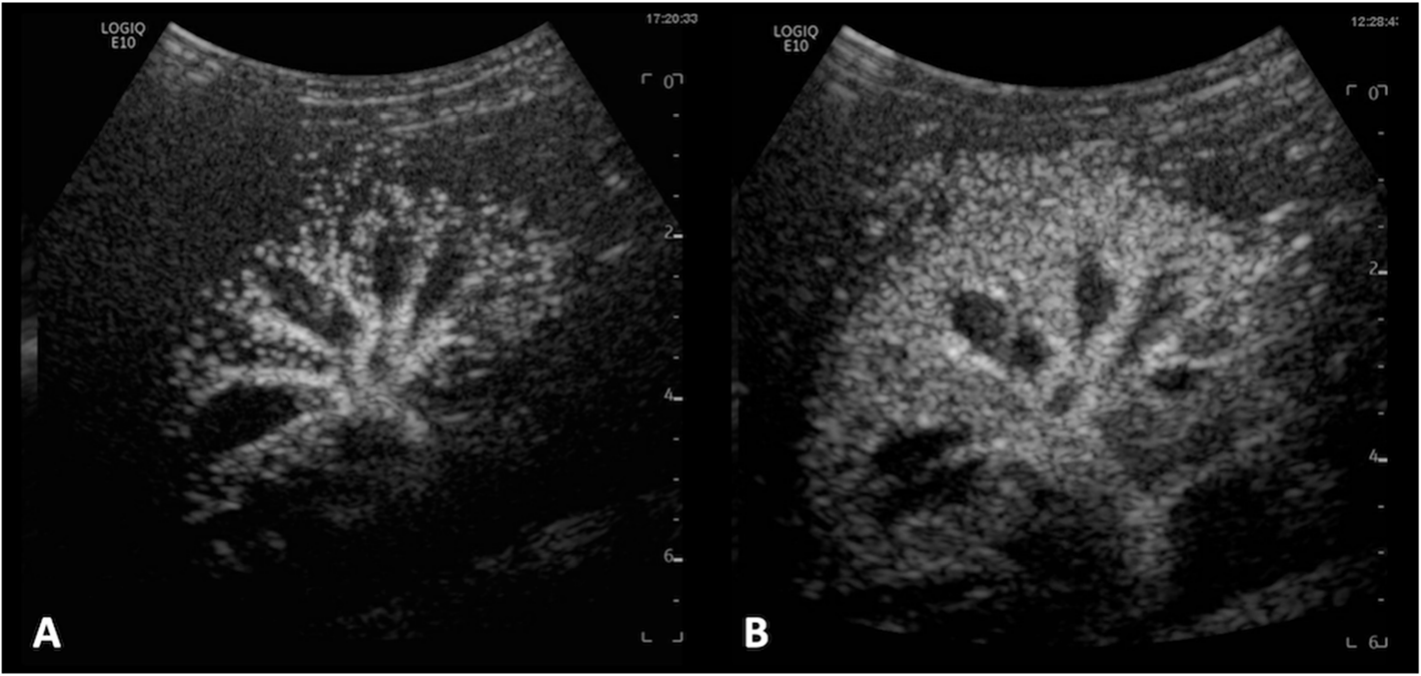
Contrast enhanced ultrasound. Single frames from a contrast enhanced US video loops taken immediately after sulfur hexafluoride lipid-type A microspheres are injected and flushed with 10mls saline. **a** Immediately pre right renal artery angioplasty with reduced cortical perfusion to the right kidney and **b** immediately post angioplasty with overall improved perfusion

Contrast enhanced US (CEUS) may play a role in evaluating renal perfusion pre and post angioplasty and may also be used as a modality for follow up. Renal CEUS can provide imaging analysis of time to parenchymal perfusion by imaging the main renal artery and branches at the hilum and identify any parenchymal perfusion defects (Fig. 3).

### Perfusion imaging

**Fig. 4:**
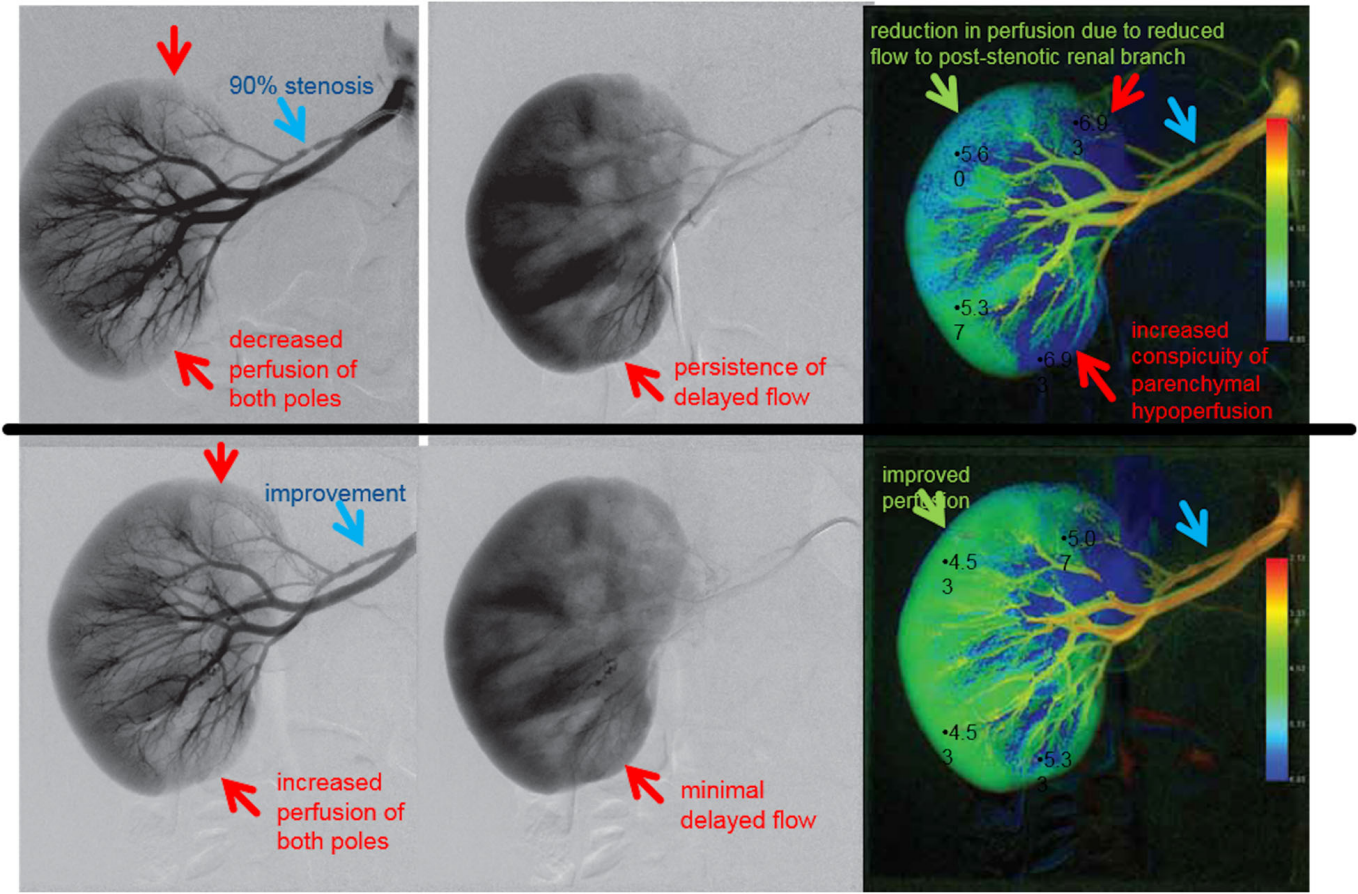
Angiographic perfusion imaging. Images of pre and post angioplasty angiography with post processing using Syngo I flow (Siemens AX) non parametric imaging data: time to peak opacification demonstrating using color coding and time points demonstrating improved time to renal perfusion post right renal accessory stenosis angioplasty

There has been growing interest recently in color-coded quantitative digital subtraction angiography (qDSA) (so called ‘perfusion imaging’). Current commercially available angiography quantification postprocessing software, such as syngo iFlow (Siemens Healthi- neers, Erlangen, Germany) and AngioViz (GE Healthcare, Chicago, Illinois) are limited by variability in flow, vessel size, amount of contrast injected, and frame rate (Patel et al. 2019). Despite this, qDSA has been reported useful in paediatric neurovascular intervention (Ma et al. 2019). At present although some centres find it a useful adjunct in renovascular intervention, its role it yet to be established (Fig. 4).

### Renal vein renin sampling

Renal vein renin sampling has been reported to be useful in children (McLaren and Roebuck 2003; Goonasekera et al. 2002). Angiographic abnormalities are often bilateral or involve small intra-renal branches. Renal vein renin sampling can lateralize and even localize an ischemic focus which is causing hypertension and help direct selective treatment (Teigen et al. 1992). Renal vein renin samples are usually taken from the infra renal IVC, each renal vein and from the upper, inter-pole and lower pole intrarenal tributaries on each side. A renin ratio of > 1.5 between the main renal veins is considered significant. A ratio of < 1.3 between the contralateral renal vein and the infra-renal inferior vena cava supports this (McLaren and Roebuck 2003; Goonasekera et al. 2002).

## Management

### Medical

Initial stabilization of the BP is undertaken using anti-hypertensive medications. Agents which block the renin-angiotensin-aldosterone system such as angiotensin-converting enzyme inhibitors (ACEi) and angiotensin receptor blockers (ARB) are contraindicated until critical main RAS or bilateral RA disease is excluded (Meyers et al. 2014). When the vascular disease is in third- and fourth-order branches, ACEi or ARB can be used to control the BP. However, the majority of children do not achieve normalization of their blood pressure despite treatment with up to six or seven different anti-hypertensive drugs (Lobeck et al. 2018; Tullus et al. 2008; Shroff et al. 2006).

### Angioplasty

Endovascular management of pediatric hypertension is indicated when blood-pressure control is inadequate or associated with significant adverse effects (Tullus et al. 2008). The goals of treatment are: restoration of renal perfusion, preservation of renal function, reduction in polypharmacy and temporizing hypertension in younger patients before surgical repair during/after puberty (Lobeck et al. 2018; Shroff et al. 2006).

Angioplasty is the most common intervention and can be performed even in small children in vessels with mild to severe stenoses. The renal artery proximal to the stenosis is used to guide angioplasty balloon size in view of the often seen post stenotic dilation distally (Fig. 1). If the balloon waist is not effaced during angioplasty balloon inflation a high-pressure balloon can then be used. The use of drug coated balloon angioplasty has been reported however there is little evidence to support their use at present (Agrawal et al. 2018). If vessels are completely occluded, they may be recanalized by angioplasty. This has been reported be successful children as small as 3.4 kg (Agrawal et al. 2018).

#### Cutting balloon angioplasty

Cutting balloon angioplasty has been used for high grade lesions resistant to conventional and high-pressure balloon angioplasty, long stenotic segments and restenosis (Srinivasan et al. 2010; Son JS 2015; Gumus et al. 2010; Alexander et al. 2017; Towbin et al. 2007). In the few series reporting cutting balloon angioplasty in children, in view of increased risk of complications such as dissection and renal artery aneurysm, cutting balloon diameter is recommended to be limited to no more than the normal vessel diameter in the incisional phase after which further dilation can be performed with a conventional balloon if required (Alexander et al. 2017; Towbin et al. 2007). Consideration should also be given to performing imaging of the arterial wall (for example by IVUS) to help place the cutting balloon in the safest position (Towbin et al. 2007).

### Stenting

Generally repeated angioplasty is considered more appropriate than stenting. Stent insertion is generally avoided in children because: stents themselves can act as a site of stenosis as the child grows, long term outcomes are unknown and in published series restenosis after stenting is significantly more common than after angioplasty (35.5% vs 17.4%) (Shroff et al. 2006; Kari et al. 2015). However, stent placement may be useful for severe or recurrent lesions, to manage iatrogenic dissection and vessels which show significant elastic recoil or restenosis after conventional or cutting balloon angioplasty (Shroff et al. 2006; Srinivasan et al. 2010; McLaren and Roebuck 2003). Surgical options should first be considered before placing a stent unless an emergent requirement. There are reports of the use of drug eluting and bioabsorbable stents in children, but this is not routine practice and there is insufficient data to support this practice (Agrawal et al. 2018; Arce-Santiago and Rodríguez-Cruz 2015).

### Ethanol embolization of ischemic foci

Superselective ethanol embolization can be used to treat diseased segmental arteries which cannot be treated by angioplasty and where open reconstructive surgery is impossible (Teigen et al. 1992; Eliason et al. 2016). Ethanol causes severe, irreversible, endothelial injury. This results in infarction of the renin-producing ischemic parenchyma with less tissue loss than nephrectomy or partial nephrectomy. Coil embolization should be avoided because occlusion of medium-sized arteries will result in recruitment of collateral supply by the ischemic focus with persistent hypertension (Tullus et al. 2008).

### Outcomes following endovascular treatment

Angioplasty (+/− stenting) for renovascular hypertension is beneficial in 48.5% to 100% of patients (Zhu et al. 2014; Shroff et al. 2006; Tyagi et al. 1997; Bayrak et al. 2008). Between 18% and 60% of patients are cured (defined as normal on no antihypertensive treatment) (Zhu et al. 2014; Lobeck et al. 2018; Shroff et al. 2006; Kari et al. 2015; Tyagi et al. 1997; Trautmann et al. 2017; Agrawal et al. 2018; Srinivasan et al. 2010; Alexander et al. 2017; Bayrak et al. 2008; Rumman et al. 2018; Colyer et al. 2014) and there is improvement (defined as BP <95th percentile while still requiring antihypertensive medications or diastolic BP reduced by more than 15% of pre-intervention) in 17–65% of patients (Zhu et al. 2014; Lobeck et al. 2018; Shroff et al. 2006; Kari et al. 2015; Tyagi et al. 1997; Trautmann et al. 2017; Agrawal et al. 2018; Srinivasan et al. 2010; Alexander et al. 2017; Bayrak et al. 2008; Rumman et al. 2018; Colyer et al. 2014).

Short-segment stenosis (< 10 mm) with residual stenosis < 10–20% after intervention have been reported to be associated with cure or clinical improvement (Zhu et al. 2014; Shroff et al. 2006; Tyagi et al. 1997; Srinivasan et al. 2010; Mali et al. 1987). The clinical result of angioplasty has not been found to be predicted by the degree of initial stenosis (Srinivasan et al. 2010) or patient age (Srinivasan et al. 2010; Alexander et al. 2017). The features predictive of outcomes are not consistent in all studies, for example one study of 28 children was unable to identify features of the diagnosis, stenosis degree or length or response to angioplasty that could predict, with statistical significance, the clinical outcome (Alexander et al. 2017).

Ostial lesions, which are more often seen in NF1 have been reported by some to be associated with better outcomes (Tyagi et al. 1997; Mali et al. 1987; Stanley et al. 1984) but not in all studies (Srinivasan et al. 2010). However, other studies in NF1 have found isolated lesions in the mid or distal part of the artery have a better outcome than lesions near the origin (Fossali et al. 2000). The presence of aneurysms, collateral vessels and mid- aortic narrowing do not predict the end result (Srinivasan et al. 2010).

The influence of underlying etiology of renovascular disease on outcomes is uncertain. Good outcomes have been reported in patients with NF1 without MAS, thought to be because these patients tend to have single, short-segment ostial stenosis and no intra-renal disease (Srinivasan et al. 2010). This conflicts with other published data that found decreased effectiveness for NF1 versus FMD and TA (Bayrak et al. 2008; Stanley et al. 1984; Fossali et al. 2000).

Despite findings that residual stenosis is predictive of poor blood pressure response, this does not always hold true. In one series 18 out of 78 treated children remained hypertensive despite radiologically adequate dilatation (Kari et al. 2015). On the other hand, a delayed response may be seen after angioplasty (Bayrak et al. 2008; Gardiner et al. 1988). The mechanism of the delayed response is thought to be related to smooth muscle spasm resulting in a false impression of residual narrowing on the post angioplasty angiogram or an increase in luminal diameter over time during arterial healing by retraction of fibrous bands (Bayrak et al. 2008; Gardiner et al. 1988).

Following angioplasty, restenosis is seen in 17 to 41% (Zhu et al. 2014; Lobeck et al. 2018; Kari et al. 2015; Tyagi et al. 1997; Bayrak et al. 2008; Colyer et al. 2014; Sharma et al. 1996; Sharma et al. 1998). Repeated angioplasty can be performed without increased risk (Shroff et al. 2006; Kari et al. 2015; Alexander et al. 2017). Reported time intervals between first and second procedures varies from 0.4 to 60 months (Kari et al. 2015). The arterial stenosis in children with renovascular hypertension is thought to be due to intimal hyperplasia (Stanley 2019). In children, non-stretch mechanisms such as tearing, flap formation, or dissection are the most common mechanisms of dilatation after balloon angioplasty (Ino et al. 1998) therefore repeated angioplasty may be advantageous in helping the vessels to remodel to a larger size.

In the long term, the success for angioplasty for FMD in children is less than that reported for adults (Srinivasan et al. 2010; Davies et al. 2008). This may be a result of smaller vessel diameter and a more pronounced response to growth factors in immature vasculature (Dillon 1997). This disease is thought to be one of fibrosis of the intima/media and not the classic medial hyperplasia described in adult females but there is limited pathological evidence for this (Stanley 2019). Other reasons for a lower response in children may be the higher rate of multiple stenosis especially those with MAS who present with widespread disease including bilateral RAS and intrarenal vascular pathology (Kari et al. 2015; Booth et al. 2002). In such cases, in the presence of significant intrarenal disease, successful treatment of main artery stenoses might fail to improve the BP (Kari et al. 2015).

Endovascular revascularization can improve renal function in addition to improving hypertension. Kari et al. (2015) reported a series of 30 children with abnormal 99 m-technetium-dimercapto-succinic acid scans (DMSA) pre angioplasty including reduced uptake or focal lesions which post angioplasty demonstrated DMSA normalization (i.e. equal uptake) in 6, improvement in 13, and static findings in 11.

#### Aortic management

In children with MAS, hypertension is often refractory to medical management despite the use of multiple medications (Zhu et al. 2014; König et al. 2006). Following angioplasty of the aorta and renal arteries +/− stenting, the change in systolic blood pressure is reported to be higher in children with mid-aortic syndrome and renal artery stenosis compared to those with isolated renal artery stenosis after intervention (Rumman et al. 2018). However, reports on long term outcomes following interventional management of mid-aortic syndrome are mixed. Some studies suggest that intervention is not very helpful with more than two thirds of children having persistent hypertension requiring long-term antihypertensive management (Rumman et al. 2018) whilst others report it very effective (König et al. 2006).

For mid-aortic syndrome, surgical interventions have been shown to require less re-intervention than endovascular interventions (Porras et al. 2013). Therefore, adopting a primary endovascular treatment strategy does require accepting that repeat interventions will be required. If planning a surgical approach, it may be preferable to wait until full adult growth and adult vascular size is reached however (Sethna et al. 2008). Adopting a primary endovascular treatment strategy may help achieve this growth*.*

### Complications

**Fig. 5:**
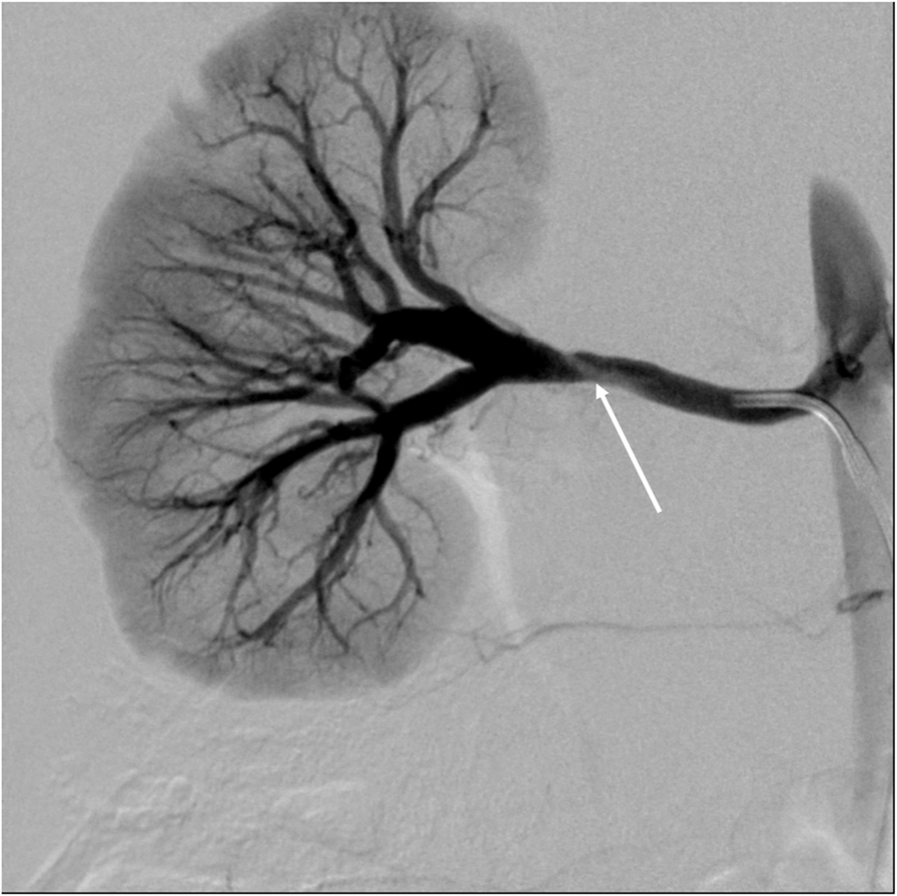
Non-flow limiting dissection. Angiography right renal artery demonstrating a lucent dissection flap with normal distal renal perfusion consistent with a non-flow limiting dissection (arrow)

**Fig. 6:**
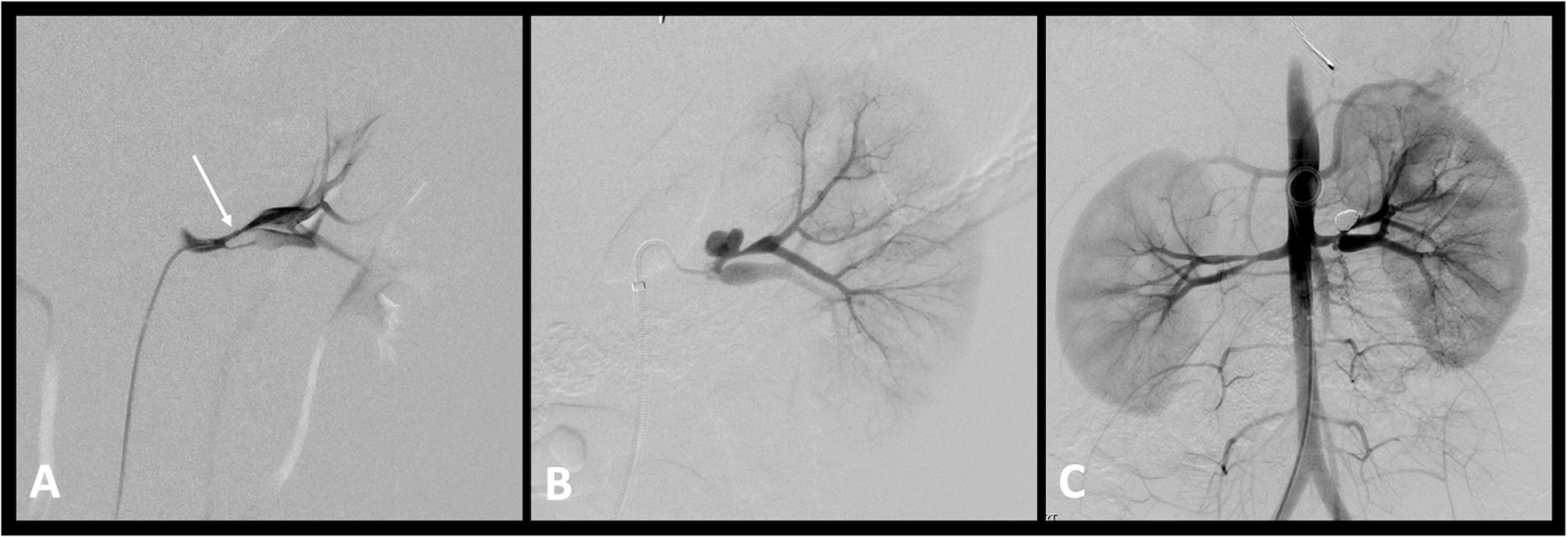
Pseudoaneursym. **a** Left renal artery angiogram demonstrating early bifurcation with tight long segment stenosis of both renal artery branches (arrow). **b** Follow up 2 month angiogram demonstrates a pseudoaneurysm at the site of angioplasty of the upper renal artery branch, treated successfully with coil embolization (**c**)

**Fig. 7:**
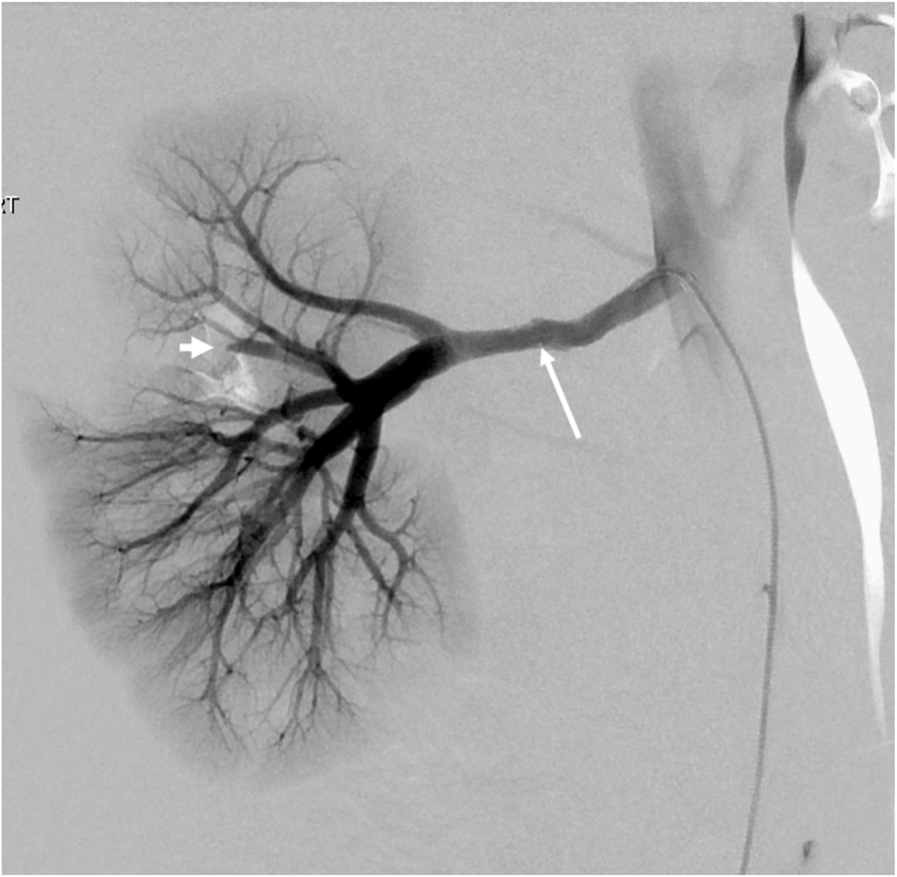
Distal embolic phenomenon. Post right renal artery angioplasty demonstrating focal segmental renal artery occlusion (short arrow) with absent perfusion consistent with a distal renal thrombo-embolic event post angioplasty (long arrow)

Reported complication rates following endovascular treatment of hypertension are reported between 0% to 43% (Lobeck et al. 2018; Srinivasan et al. 2010; Alexander et al. 2017). Complications include contrast-induced nephropathy, arterial spasm, dissection (Fig. 5), delayed pseudoaneurysm (Fig. 6) formation and perforation. Parenchymal perfusion defects can occur with distal embolic phenomena post angioplasty (Fig. 7) or in situ thrombosis from prolonged guidewire lodgement in segmental vessels. Local dissection happens often and is expected, especially with the use of the cutting balloon, and as discussed above may be involved in the process of vascular remodelling and are not usually hemodynamically significant (Fig. 5) (Tullus et al. 2008). Procedure related deaths are rare. Kari et al. (2015) reported one procedure-related death in a series of 114 procedures. This was secondary to haemorrhage from a synthetic graft tear. In the event of arterial rupture if balloon reinflation or covered stent placement is not possible vascular surgery consultation should be readily available (Srinivasan et al. 2010; McLaren and Roebuck 2003). Following endovascular treatment the BP pressure may be labile and can increase the risk of stroke in children with concomitant cerebrovascular disease (Tullus et al. 2008), therefore assessment of the head and neck vessels should be considered prior to intervention (Sethna et al. 2008). Accelerated or worsening of hypertension following angioplasty has also been seen as rare complication (Srinivasan et al. 2010; Larar and Treves 1993).

## Surgery

In adults, a meta-analysis including 47 angioplasty studies (1616 patients) and 23 surgery studies (1014 patients) has shown the cure rate of surgery is slightly higher (54%) than that of angioplasty (36%) however this is at the cost of two and a half times higher major complication rate post-surgery compared to angioplasty (15% vs. 6%) (Trinquart et al. 2010). Similarly in a retrospective study including 381 patients, surgery for renal artery stenosis has been shown to have a higher complication rate and higher early and long term mortality (Alhadad et al. 2004). There is a paucity of similar data in the paediatric population.

Surgery is used infrequently as a primary treatment in children in many centres. Lobeck et al. (2018) reported that 10 patients in their series of 39 patients treated over 21 years underwent primary surgery. Reports from large series of open surgical interventions have high rates of cure of 70% (Stanley et al. 2006) and 82% (Lacombe 2011). Primary surgical intervention with a goal of managing renovascular disease in a single operation is not typically performed in view of the technical challenges in small children. There is some surgical viewpoint that angioplasty may alter the typical surgical approach, converting a primary renal arterial re-implantation into an aorto-renal bypass due to secondary fibrotic changes post angioplasty (Eliason et al. 2016). Studies in adult patients have shown that renal angioplasty has a shorter time to first re-do than surgical revascularization for renovascular disease (Alhadad et al. 2004). Given the established safety and success of endovascular intervention, at most institutions it remains the preferred treatment option.

## Conclusion

Current literature supports an endovascular approach for investigation and management of renovascular hypertension in children. However, the outcome data presented here is derived from retrospective or prospective observational studies on relatively small patient numbers over long time periods with heterogeneity of reported cohorts in terms of etiology, response to medications, vascular involvement and endovascular treatments used (Zhu et al. 2014; Lobeck et al. 2018; Shroff et al. 2006; Kari et al. 2015; Agrawal et al. 2018; Srinivasan et al. 2010; Alexander et al. 2017; Bayrak et al. 2008; Rumman et al. 2018; Colyer et al. 2014; Mali et al. 1987; Stanley et al. 1984; Peco-Antić et al. 2016). The wide ranges of reported outcomes and paucity of information regarding factors predictive of outcomes relates to the nature of published studies. It is therefore difficult to develop clear guidelines for the diagnosis and management of renovascular hypertension in children. Prospective randomized studies are unlikely to play a significant role due to the rarity of the pathology and heterogeneity of the population.

In the future, data from national registries such as the United States Registry for FMD which was created in 2008 (Green et al. 2016) and dedicated working groups such as those formed at the First International Symposium on Pediatric Renovascular Hypertension in Ann Arbor, Michigan in November 2019, should help to develop more robust guidelines. At present there is consensus agreement that children with renovascular hypertension should be managed at centres with expertise in this area and the availability of multi-disciplinary care including, paediatric nephrologists, paediatric vascular surgeons and a paediatric interventional radiology team.

## Data Availability

Not applicable.

## Abbreviations

IR: Interventional Radiology
US: Ultrasound
RVH: Renovascular hypertension
FMD: Fibromuscular dysplasia
TA: Takayasu’s arteritis
CTA: CT angiogram
MRA: MR angiogram
DMSA: 99 m-technetium-dimercapto-succinic acid
IVUS: Intra-vascular ultrasound
OCT: Optical coherence tomography
